# An Analysis of Predictive Sample-to-Cutoff Index for HIV Infection Confirmation Using Elecsys® HIV Combi PT Assay

**DOI:** 10.1155/2022/5097189

**Published:** 2022-08-10

**Authors:** Xiaohong Xia, Xiang Zhang, Jun Zhou, Mengying Zhang

**Affiliations:** ^1^Department of Laboratory Medicine, Branch of National Clinical Research Center for Laboratory Medicine, The First Affiliated Hospital of Nanjing Medical University, Nanjing 210029, China; ^2^Branch of National Clinical Research Center for Laboratory Medicine, Nanjing 210029, China; ^3^Department of Infection Management, The First Affiliated Hospital of Nanjing Medical University, Nanjing 210029, China

## Abstract

**Background:**

Early and rapid diagnosis is crucial in HIV preventing and treatment. However, the false-positive rate (FPR) by 4-th generation detection assays was high in low-HIV-prevalence regions.

**Objectives:**

To analyze the relation between sample-to-cutoff index (COI) and HIV confirmatory results, and to explore a new COI threshold in our own laboratory to predict HIV infection.

**Methods:**

We retrospectively analyzed primarily reactive results by Elecsys® HIV combi PT assays and their confirmatory results by western blot (WB) at Nanjing Center for Disease Control and Prevention (CDC). The mean COI values of true positive (TP), false positive (FP), and indeterminate groups were compared, and receiver operating characteristic curve (ROC) analysis was performed to determine the optimal COI value for predicting HIV infection.

**Results:**

Totally 150,980 HIV serological results were reviewed, and 305 (0.2%) were primarily reactive. There are 82 (26.89%) true positives, 210 (71.92%) false positives, and 11 indeterminate samples confirmed by WB tests, and another 2 patients rejected WB tests. Mean COI values of TP (643.5) were greatly higher than that of FP (3.174) (*P* < 0.0001), but there is no significant difference between FP and indeterminate groups. Combining the requirement of HIV diagnosis and ROC analysis, 9.87 was established as the optimal threshold to predict the infection, with 100% sensitivity and 99.99% specificity.

**Conclusions:**

By adjusting the COI threshold, the FP samples can be reduced and the efficiency of screening assays can be increased, which can save much additional reagent and staff costs and much time for delivery of HIV test results.

## 1. Introduction

Human immunodeficiency virus (HIV) infection, which can progress to acquired immune deficiency syndrome (AIDS), is becoming a global pandemic [1]. It was reported that the number of people living with HIV/AIDS (PLWH) in China at the end of 2014 is nearly 501,000 [2]. Most recent estimation results indicated that the number was already beyond one million by the end of 2018 and would keep growing, causing much pressure of HIV/AIDS prevention and control [3]. Compared with established infections, new and recent HIV infections are more infectious. And acute infection contributes significantly to HIV transmission, accounting for 10% to 50% of new cases [4–9], and usually the median time interval between infection and diagnosis is 3.3 years [10]. Nearly 40% of PLWH are unaware of their HIV status, which greatly increases the potential risk of transmission. Thus, early and rapid identification of HIV infection is crucial to prevent its further transmission, and HIV testing algorithms aim to minimize the time between infection and confirmatory diagnosis with maximum sensitivity and specificity [11–13]. Simultaneously detecting the HIV-1/2 antibodies and the P24 antigen, 4th-generation assays allow for more sensitive detection of early acute HIV infection cases and reduction of the window period by 4 to 8 days compared with 3rd-generation assays, which only detect HIV antibodies [14–18]. Hence, in current diagnostic algorithms, 4th-generation assays are gradually replacing 3rd-generation assays and being implemented worldwide as screening tests [19]. Elecsys® HIV combi PT assays (Roche Diagnostics GmbH, Penzberg, Germany) are performed to screen HIV infection, and the results are showed as sample-to-cutoff index (COI), which is calculated automatically by comparing the chemiluminescence signal magnitude of the sample with the cutoff value determined by calibration. According to the manufacturer's specification, samples are defined as reactive if the COI ≥1.0, and nonreactive when COI is less than 0.9. Samples (0.9 ≤ COI < 1.0) were defined as borderline cases, processed as reactive specimens [20,21]. Despite having higher sensitivity, 4th-generation assays are found to have a higher false-positive rate (FPR). Due to a variety of factors, weakly positive COI results are commonly observed and often followed by a nonreactive or indeterminate confirmatory test result in uninfected population [22–25]. It is difficult to differentiate early seroconversion from false positives, especially for those slightly positive samples. In our hospital, according to the Chinese Centers for Disease Control and Prevention (CDC) guideline, a specimen with COI ≥0.9 has to be retested after long and high-speed centrifugation. When the retest result is still reactive, a different method test should be used before submitting for confirmation at Nanjing CDC. These primary and confirmatory tests significantly largely increase reagent and staff costs and also slow the delivery of the patient's test results to the clinician. In addition, HIV false-positive results may cause inappropriate anxiety to patients or even undesirable consequences [13].

Recently, many studies show that there was an association between test values and subsequent confirmatory results. As COI values increased, the number of FP decreased, and the possibility of positive results was higher [13, 26, 27]. Thus, the present study was carried out to evaluate the performance of Elecsys® HIV combi PT assay in a large cohort of patients from 2018 to 2019 in Nanjing, a low-HIV-prevalence region of East China, to analyze the sensitivity, specificity, and positive predictive value (PPV) at different COI, and to seek for an optimal cutoff that can predict HIV infection at early period.

## 2. Materials and Methods

### 2.1. Study Population

We retrospectively collected testing episodes from the First Affiliated Hospital of Nanjing Medical University between January 2018 and January 2019. A total of 150, 980, including all repeatedly reactive tests, HIV screening results were analyzed by Elecsys® HIV combi PT assays. All the data were from the laboratory information system (LIS) and hospital information system (HIS) of the First Affiliated Hospital of Nanjing Medical University.

### 2.2. HIV Diagnostic Algorithm

**Figure 1:**
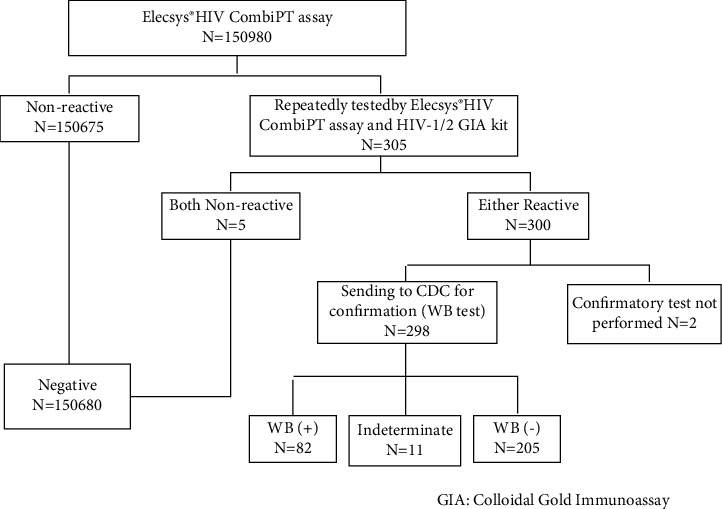
HIV diagnostic algorithm and sample results distribution.

All specimens were centrifuged for 10 minutes at 3500 rpm and then detected by Elecsys® HIV combi PT assay on the Cobas e601 analyzer (Roche Diagnostics GmbH, Mannheim, Germany). Any sample with COI ≥0.9 was retested in duplicate after 10000 *g*, 10-min centrifugation. For samples still reactive, a 3rd-generation gold-labeled immune assay (GIA) kit, XinChuang HIV-1/2Ab (InTec, INC, Xiamen, China), was used to perform the supplemental tests for initially screening reactive cases. Initially, screening reactive cases should be submitted for confirmatory tests (WB, nucleic acid, or p24 antigen tests). Currently, western blot (WB) analysis (HIV1/2 BLOT 2.2; MP Biomedicals, Singapore) is taken for confirmation at Nanjing CDC; nucleic acid and p24 antigen tests were not applied (Figure 1). Thus, for the non-positive by WB, timely follow-up (week 2, week 4, month 3, and month 6) is required for HIV infection exclusion. According to the Chinese CDC criteria [28], HIV WB results interpretation is as follows: HIV-1 Positive: presence of at least two *env* bands plus one *gap* or one *pol* band; Indeterminate: reactive to any specific band but not compatible with positive criteria; Negative: absence of any of the specific bands; and HIV-2 Positive: presence of *gp36* band.

### 2.3. Statistical Analysis

The continuous variables were expressed by mean, and multiple groups were performed using the one-way ANOVA test and Kruskal–Wallis test. We use the receiver operating characteristic curve (ROC) analysis to determine the optimal cutoff value for predicting true positives (TPs). All statistical analyses were two-sided, and *p* value < 0.05 was considered statistically significant. GraphPad Prism 7.0 (GraphPad Software, San Diego, CA, USA) was used for data analysis.

## 3. Results

### 3.1. Testing Episodes

During the study period, a total of 150,980 specimens were tested by using Elecsys® HIV combi PT assay, including 150,675 patients negative and 305 patients initially reactive (Figure 1). 5 tests were negative and 300 were repeatedly reactive after centrifugation (10000 g, 10 min) and HIV-1/2 GIA kit testing. In all 300 repeatedly reactive cases, 82 of 298 cases were found positive, 205 of 298 were negative, and 11 of 298 were indeterminate after WB tests and follow-ups. There are 2 patients who refused to WB test and were lost to follow-up. Overall, according to the HIV identification algorithm in our hospital and Nanjing CDC, HIV prevalence in this study is 0.054% (82/150,980), largely consistent with previous reports in general Chinese population [29], and 82 (29%) and 205 (71%) of 287 cases were identified as true positives (TPs) and false positives (FPs), respectively. The positive rate (PR) of males (63/82) was 76.8%, much higher than 23.2% of females (19/82). False-positive cases accounted for 0.10% of the total.

### 3.2. COI Distribution

**Figure 2:**
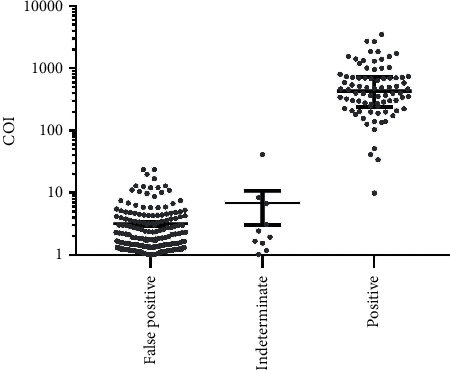
The mean COI values of each group.

The COI of all samples ranged from 0.1 to 3523, the highest COI for true-negative (TN) samples was 23.77, and the lowest COI for TP was 9.95. As shown in Figure 2, the mean COI values in TP (643.5) were significantly higher than that in FP (3.17) (*p* < 0.0001) and indeterminate samples (IN) (6.839) (*p* < 0.0001), while the difference of mean COI in the IN group and FP group was not statistically significant.

There were 10 kinds of WB bands patterns in all 82 WB-positive specimens (Table 1). No relation between WB bands number and COI values was observed, which is different from the previous study by Wang et al. [30].

### 3.3. ROC Analysis

ROC analysis showed that the sensitivity and specificity were 100% and 99.86%, respectively, when COI >1.0, while the FP number was up to 210. And no loss of sensitivity was observed until the COI threshold was raised to 9.87, while the FP number can be reduced to 14.  Table 2 showed different sensitivity, specificity, false-positive situation, and PPV at different COI values. For COI threshold values of 9.87 and 12.79, the sensitivity can achieve 100% and 98.78%, with much lower FP number (14 and 6, respectively). When the COI was raised to 37.75, it can achieve 100% of specificity, while the sensitivity was decreased 97.56%. Considering the clinical utility, 9.87 was recommended as the optimal COI by ROC (with 100% sensitivity and 99.99% specificity).

## 4. Discussion

Early diagnosis of HIV infection is necessary to facilitate prompt treatment and prevent further transmission, as new and recent infections are more infectious than chronic infections [4]. The Global Health Sector Strategies on HIV (2016–2021) have proposed that 90% of PLWH can be tested [31]. Only 68% of PLWH were diagnosed in China, although it is low HIV prevalence [32]. Hence, HIV screening assay must be highly sensitive and can detect all HIV subtypes. Currently in China, few of new HIV infection can be detected at early period due to rare application of 4-th generation assay and nucleic acid (NAT) and p24 antigen tests in many hospitals [30]. In addition, 4-th generation assay with high sensitivity has a high false-positive rate (FPR = 0.14% in our study), which requires many retests and confirmatory tests subsequently according to the current diagnostic algorithm. These retests and confirmatory tests significantly increase reagent and staff costs, slowing the transmission of the test results to the clinician and further treatment. And it also caused many patients unnecessary anxiety when waiting for the confirmatory results. It was reported by many studies that HIV serology COI values were strongly correlated with the probability of confirmed HIV infection [11,12]. Thus, understanding the performance of fourth-generation assay and establishing a predicted threshold COI for HIV positive results in a local region were pivotal in clinical practice.

The present study retrospectively analyzed 150,980 specimens performed by Elecsys® HIV combi PT assay, in which the prevalence of HIV was 0.054%. Although the 4-th generation Elecsys® HIV combi PT assay was reliable for screening of HIV with high sensitivity (100%) and specificity (99.86%), it resulted in a high false-positive rate (FPR) (0.14%) in Nanjing, close to other studies in the low-HIV-prevalence setting [19]. The mean COI of TP (643.5) was significantly higher than that of FP (3.17). And as COI values increased, the PPV for HIV infection also went up, while FPR decreased. In our study, when COI values were at 1.00, 9.87, and 37.75, the PPV was 28.08%, 85.42%, and 100%, respectively, while FP number was 210, 14%, and 0, respectively. Previous reports have proposed different predicted COI thresholds, while it varied depending on different test assays, patient population, local prevalence, and technical factors [11,19,27,33]. Due to such differences, each institution should have its own COI threshold values. According to the ROC analysis of our study, the COI value with 100% PPV for HIV confirmation was ≥37.75. While considering the requirement of HIV diagnosis for sensitivity, we recommended 9.87 as the optimal COI threshold, which provided a sensitivity and specificity of 100% and 99.99%, respectively, and eliminated 196 FP cases.

Actually, for HIV diagnosis protocol, follow-ups and WB from CDC are still be used as mainly confirmatory tests according to legislation. However, in view of high false-positive rate of HIV screening, which led to great waste of medical resource, we suggest to raise the threshold properly so that the false-positive rate can be reduced in low-HIV-prevalence regions like Nanjing. Also, the COI value can be used to predict infection for those specimens with high COI value so that the clinicians can make some timely and appropriate measures before the confirmatory results, not just waiting for CDC's feedback. The prediction can also help relieve patients' depression and anxiety.

The limitation of this study involves some unidentified HIV infection status due to refusing WB test and some indeterminate WB results. Most of these patients were lost to follow up, and only 3 indeterminate patients were in contact and found to be positive subsequently. And the nucleic acid test (NAT) and p24 antigen test are also not applied at Nanjing CDC and our hospitals, which resulted in hardness to knowing some seroconversion or acute HIV infection and needed more detailed follow-up. Then, the study was operated in one single medical center, and hence the established COI threshold cannot be applied by other institutions due to differences in patient and technical factors; therefore a multicenter study is required.

In summary, this study showed that the Elecsys® HIV combi PT had excellent sensitivity, but high percent of false-positive results led to various problems. An optimal COI value is necessary for predicting the final status of HIV infection, rapidly and preliminary reportingHIV infection prior to a confirmatory result. In our hospital, COI ≥9.87 was recommended to predict HIV infection at early period, to make up for the lack of high FPR of the 4-th generation HIV screening test.

## Figures and Tables

**Table 1:** WB bands analysis.

No. of bands	WB bands pattern	No. of samples	COI (Mean)
All (10)	gp160 gp120 p66 p51 gp41 p39 p31 p24 p17 p55	41	594.74
9	gp160 gp120 p66 p51 gp41 p39 p31 p24 p17	10	540.78
	gp160 gp120 p66 p55 p51 gp41 p31 p24 p17	2	
8	gp160 gp120 p66 p55 p51 gp41 p31 p24	3	986.86
	gp160 gp120 p66 p51 gp41 p31 p24 p17	15	
7	gp160 gp120 p66 p51 gp41 p31 p24	7	573.36
	gp160 gp120 p51 gp41 p31 p24 p17	1	
	gp160 gp120 p66 p51 gp41 p24 p17	1	
5	gp160 gp120 gp41 p31 p24	1	1403
4	gp160 gp120 gp41 p24	1	703.6

**Table 2:** Sensitivity and specificity at different cutoffs.

	Values at different cutoffs				
	1.00	5.94	9.87	12.79	37.75
Samples ≥COI	292	104	96	87	80
FP samples	210	22	14	6	0
Eliminated FP	—	188	196	204	210
TP samples	82	82	82	81	80
Sensitivity (%)	100	100	100	98.780	97.561
Specificity (%)	99.86	99.985	99.991	99.996	100
PPV (%)	28.08	78.85	85.42	93.10	100

## Data Availability

The data used to support the findings of this study are available from the corresponding author upon request.
